# Supplementary material for: Paleolatitude.org 3.0: A calculator for paleoclimate and paleobiology studies based on a new global paleogeography model
Source: PLoS One. 2026 Apr 29;21(4):e0346817. doi: 10.1371/journal.pone.0346817 (PMC13127928; doi:10.1371/journal.pone.0346817)
Supplement: S1 Data — Supplementary Files: A set of supplementary files is available at DOI: https://doi.org/10.6084/m9.figshare.31021144, containing the following elements: S1 File. GPlates files (www.gplates.org [15]) of the Utrecht Paleogeography Model presented in the paleogeographic maps of Figs 1 and 3, and the rigid polygon version that is used as basis for the Paleolatitude.org tool. These consist of a rotation file, and a series of shape files (in gpml format) that underpin the paleogeographic model, a gpml file of the rigid polygons that are used to rotate coordinates in the Paleolatitude.org tool, as well as a project file (gproj) of the entire paleogeographic reconstruction. S2 File. Details of gAPWP25. In addition to a Readme.txt file with general descriptions and the gAPWP25.rot file with the updated paleomagnetic reference frame in GPlates rotation format, the files contain: S1 Table. Changelog of the update of gAPWP23 to gAPWP25. S2 Table. Paleomagnetic database used to compute the global apparent polar wander path for the last 320 Ma. We have listed age constraints, statistical parameters, Euler rotation parameters and other metadata per paleomagnetic pole used in the parametric re-sampling scheme. For more details, see main text. The grey-colored entries are excluded from the computation of the APWP. See columns ‘age constraints’, ‘comments’ and ‘reliability’ for specific details for a given dataset. Abbreviations: min_age and max_age = lower and upper boundaries of age uncertainty range; slat/slon = latitude and longitude of (mean) sampling location; N = number of paleomagnetic sites used to compute the paleopole; mDec/mInc = mean declination of inclination; α95/A95 = radius of the 95% confidence circle about the mean of the distribution of directions/VGPs; k/K = Fisher [109] precision parameter of the distribution of directions/VGPs; plat/plon = paleopole latitude and longitude (south pole); K_est/A95_est = values estimated using formula of Cox [214] (eq. 24); plateID [file pone.0346817.s001.zip › Supplementary information_vanHinsbergen_PLoSOne_2026/Paleolatitude_API_Manual.pdf]

Paleolatitude API Manual

API Endpoint: `https://paleolatitude.org/api/paleolatitude/lat/lon/age/minAge/maxAge/model`

What is a REST API?

A REST API is a way to request data from a server using a web address (URL), similar to visiting a website. Instead of getting a web page, you get data back (usually in JSON format) that your program can use. You can test API requests directly in your web browser or use tools like Excel, Python, R, or specialized applications like Postman.

How to Use This API

To get paleolatitude data, you construct a URL with values in a specific order: latitude, longitude, age, minimum age, maximum age, and model.

URL Structure

```
https://paleolatitude.org/api/paleolatitude/lat/lon/age/minAge/maxAge/model
```

Required Parameters

- **`lat`** - Latitude of your site in decimal degrees (-90 to 90)
- **`lon`** - Longitude of your site in decimal degrees (-180 to 180)
- **`model`** - The paleomagnetic reference frame to use (see available models below)

Optional Parameters

- **`age`** - A specific age in millions of years (Ma) to calculate paleolatitude for (use -1 if not needed)
- **`minAge`** - Start of age range in Ma (minimum: 0, use 0 if not needed)
- **`maxAge`** - End of age range in Ma (must be minAge, use 9999 if not needed)

Important: If you specify an `age`, it must fall between `minAge` and `maxAge`.

Example Requests

Example 1: Single Point in Time

To get the paleolatitude of a site at 50°N, 10°E at 100 Ma using a specific model:

```
https://paleolatitude.org/api/paleolatitude/50/10/100/0/9999/vaes
```

Example 2: Time Series

To get paleolatitude data for the same site from 50 to 150 Ma:

`https://paleolatitude.org/api/paleolatitude/50/10/-1/50/150/vaes`

Example 3: Specific Age Within a Range

To get data at 100 Ma but with uncertainty bounds calculated from 95-105 Ma:

`https://paleolatitude.org/api/paleolatitude/50/10/100/95/105/vaes`

Understanding the Response

The API returns data in JSON format with the following structure:

```json
{
  "lat": "50.0",
  "lon": "10.0",
  "plate": {
    "id": "315",
    "name": "Eurasia"
  },
  "paleolatitude": [
    {
      "age": 100.0,
      "lat": 42.15522,
      "lowerbound": 40.08026,
      "upperbound": 44.33417,
      "interpolated": true,
      "baseplate": "South Africa (701)"
    }
  ]
}
```

Response Fields:

- **`lat`** / **`lon`** - Your input coordinates
- **`plate.id`** - Tectonic plate ID for your site
- **`plate.name`** - Name of the tectonic plate
- **`paleolatitude`** - Array of paleolatitude data points:
  - **`paleolatitude[..].age`** - Age in Ma
  - **`paleolatitude[..].lat`** - Calculated paleolatitude in decimal degrees
  - **`paleolatitude[..].lowerbound`** / **`upperbound`** - Uncertainty bounds (95% confidence)
  - **`paleolatitude[..].interpolated`** - Whether this point was interpolated between two known points in the calculation model (true/false)

- `paleolatitude[..].baseplate` - Reference plate

Available Models

The `model` parameter specifies which paleomagnetic reference frame to use. The following four models are currently supported:

- **`vaes`** - Vaes et al. (2023)
- **`torsvik`** - Torsvik et al. (2012)
- **`besse-courtillot`** - Besse & Courtillot (2002)
- **`kent-irving`** - Kent & Irving (2010)

Error Messages

If your request has invalid parameters, you'll receive an error message explaining the problem:

- **"Latitude must be a number between -90 and 90 degrees"**
- **"Age must be set between min age and max age"**
- **"Given paleomagnetic reference frame does not exist"**
- **"No plate found for site"**
- **"The provided site is located on an unconstrained plate"** - Model lacks data for this plate
- **"Insufficient model data available"** - No data for the requested time period

Tips for Use

1. **Age precision** - Ages are automatically rounded to 1 decimal place
2. **Default ranges** - If you only specify `age` without min/max, you'll get just that single time point
3. **Browser testing** - You can paste any example URL directly into your browser's address bar to see the results

Using the API in Different Tools

In a web browser: Simply paste the URL with parameters into the address bar.

In Python:

```python
import requests
url = 'https://paleolatitude.org/api/paleolatitude/50/10/100/0/9999/vaes'
response = requests.get(url)
data = response.json()
```

In R:

```r
library(httr)
library(jsonlite)
url <- "https://paleolatitude.org/api/paleolatitude/50/10/100/0/9999/vaes"
response <- GET(url)
data <- fromJSON(content(response, "text"))
```

In Excel (Power Query): Use Data → Get Data → From Web and enter the URL with parameters.
